# Effects of Sibship Size and Birth Order on Sexual and Reproductive Health among Sexually Active Young People in China

**DOI:** 10.3390/children9091302

**Published:** 2022-08-27

**Authors:** Luoqi Yuan, Wenzhen Cao

**Affiliations:** 1 School of Economics, Peking University, Haidian District, Beijing 100871, China; 2 School of Public Health and Management, Wenzhou Medical University, Chashan University Town, Wenzhou 325035, China

**Keywords:** sibling effect, birth order, only children, sexual and reproductive health, young people

## Abstract

Only children are more prevalent among young people today in China due to the globally renowned one-child policy since the 1980s, but the association between sibship size and the sexual activity of youth needs to be further clarified. The aim of this study was to explore the effect of siblings, being an only child, and birth order on the sexual and reproductive health (SRH) of young people. Data were utilized from 11,044 sexually active college/university students who participated in a large-scale national survey. Overall, numerous undergraduates nationally identified as only children (43.5%); for non-only children, 32.4% were oldest children, 10.5% were middle children, and 13.6% were youngest children. For both sexes, having more siblings was related to having risky sexual debuts and less contraceptive use. Furthermore, young men and young people born in rural areas with more siblings were more likely to have severe health outcomes, such as unwanted pregnancy and sexually transmitted infection(s). Finally, being an only child protected youth from risky sexual behaviors and adverse health outcomes. For students with siblings, middle children were more inclined to risky sexual initiation and low frequency of contraception compared to first-borns. Our analysis provides the first evidence of one child and sibling effects on SRH in China and has significant implications for promoting SRH in the context of encouraging childbirth.

## 1. Introduction

Improving sexual and reproductive health (SRH) is a global consensus [1], and risky sexual behavior causes significant disease burdens in both developed and developing countries [2]. For sexually transmitted infections, according to the World Health Organization (WHO), more than one million people worldwide become newly infected every day [3]. Additionally, risky sexual behaviors can lead to infertility, reproductive system diseases, and unintended pregnancies [4]. As of today, there are 1.2 billion young people between the ages of 15 and 24, representing 16% of the population of the world [5]. Unsafe sex and lack of contraception are the main risk factors for incident disability adjusted life years (DALYs) for young people [6].

The previous research on the determinants of SRH focused on the organization of the health system and social conditions. Common determinants related to the health system include the role of providers [6,7] and service delivery [8,9,10]. Social conditions include poverty [11,12], migration [13,14,15], school-based education [16,17,18], and sexual violence or coercion [19,20]. As family is a critical context for young people to learn norms and behaviors of SRH, some studies have also paid attention to the relationship between family and SRH, such as the role of parents [21,22,23]. However, only limited studies supported the sibling effect, such as birth order [24,25], older siblings [26], or sibling similarity [27,28], and with mixed conclusions.

As siblings compete for attention and resources from parents, different family niches develop for children with different numbers of siblings [29,30]. On the one hand, only children are not exposed to sibling rivalry and receive parental investment naturally. The existing literature suggests that children with more parental attention are more likely to have positive attitudes towards family compared to those receiving less parental attention [29,30]. On the other hand, children may avoid risky sexual behaviors through the sibling effect. Older siblings serve as more efficient mentors to provide knowledge on safe sex and set norms of conduct [24,31,32], and conversations about sexuality between siblings also help in learning about sexual activities [31,32,33]. Considering birth order, oldest children are born without sibling rivalry and experience early individual development as only children, so they are more conscientious and responsible than younger children [34,35]. Moreover, oldest and youngest children are more likely to regard family resources as a dependable support, compared to middle children [30,36].

In China, university is usually a different stage for young people, where they are exposed to peers from diverse cultures, have less academic pressure compared to high school, and have fewer parental and school restraints. Under profound socioeconomic changes in China, youth take less conservative attitudes towards premarital sex and are gradually becoming more sexually active. The data from the same national survey used in this study show that college students have a high degree of acceptance of premarital sex: only 11.4% made it clear that premarital sex was not acceptable; and by the time college students entered senior year, more than half of the students (52.9%) had already experienced penetrative sex [37]. However, the adverse health outcomes due to poor knowledge of SRH, such as sexually transmitted infections (STIs) and unwanted pregnancy, have caused physical and psychological problems among Chinese young people and led to severe burdens on society as a whole. Surveys show that 23% of sexually active unmarried women had experienced unintended pregnancy, and nearly 20% of young women who had an induced abortion had undergone repeated abortion [38].

Studies investigating the determinants of SRH in China mainly focused on sexual knowledge, attitudes towards sex, socioeconomic status, and lifestyles. These finding based on all Chinese young people may not be applicable to sexually active populations at high risk for sexual and reproductive health [13,17,39,40,41]. In addition, the one-child policy of China is world-renowned, and only children are more common among the teenage generation of today than ever before. Whether being an only child and the number and rank order of siblings have associations with SRH remains unknown in China. Therefore, the objective of this study was to analyze the relationships between sibling numbers, being an only child, and birth order with SRH among Chinese young people, which will allow us to better promote SRH among young Chinese people. More importantly, we extended the sibling effect on SRH to sibship size and birth order, while the current literature has largely focused on and considers one-child status as a dichotomous construct [39,42,43].

## 2. Materials and Methods

### 2.1. Data Source

#### 2.1.1. Questionnaire Survey

The data for the current study were collected as part of the project “2019–2020 National College Student Survey on Sexual and Reproductive Health” (NCSS-SRH) commissioned by the China Family Planning Association (CFPA), whose objective was to provide representative data to describe the overall prevalence and characteristics of knowledge, attitudes, and practices (KAP) regarding SRH among Chinese college/university students. The large national complex survey was conducted through Questionnaire Star (https://www.wjx.cn, accessed on 11 June 2022), a professional online survey service that potential participants throughout China can easily access. Cookie-based duplication protection was adopted to automatically prevent repetitive responses. All participants were voluntary and provided fully informed consent. Data collection was anonymous without any individual login code or personal identifier. Regardless of time and space, the survey questionnaire could be administered through the personal smartphones of respondents (the primary approach), tablets, desktop computers, or laptops. Data collection was approved by the Institution Review Board of Tsinghua University (#20190083). Details regarding the study design and sampling frame have been reported in depth elsewhere [44,45].

#### 2.1.2. Study Subjects

Due to the snowball effect of social media promotion and on-campus publicity, 55,757 college/university students completed the online survey by the close of survey. Firstly, this study excluded samples that were not in college enrollment status at the time of the survey, those that provided invalid informed consent, and those that failed to pass quality control questions such as attention check questions, resulting in 54,580 participants with high-quality responses. Then, the respondents that entered the final analyses were limited to: (1) those who were undergraduates, excluding a small number of those who were studying postgraduate degree programs (as the project was conducted primarily among undergraduates); (2) those whose habitual residence was not in an overseas area or a foreign country prior to university admission; and (3) considering that this study used multiple variables related to sexual behavior and contraception, all analyses were conducted among sexually active university students (defined as previously engaging in penetrative sex). The final sample consisted of 11,044 qualified college/university students with a broad geographical distribution of all 31 provincial administrative regions in China.

### 2.2. Measures

#### 2.2.1. Sibship Size, Only Child, and Birth Order

All respondents were asked initial questions to evaluate sibship size: “how many siblings did you live with when you were growing up in your family?” Participants who reported having no siblings were considered “only child.” For young people with siblings, a further question was asked: “what is your ordinal position among your siblings?” According to the responses to this question, birth order was categorized into three groups: oldest (i.e., first-born) child, middle (i.e., middle-born) child, and youngest (i.e., last-born) child. The middle child was defined as a child who had at least one older and at least one younger sibling.

#### 2.2.2. Outcome Variables

Sexually active participants (those who reported vaginal or anal sex) were further asked whether they had engaged in particular behaviors or situations: sexual initiation (SI) after drinking alcohol, SI after using pornography, unprotected first intercourse, unintended pregnancy, and diagnosed STI(s). With regards to STI(s), it was explicitly asked if the respondents had ever been medically diagnosed with at least one type of STI. These variables were recoded into binary variables. Participants reported their age at their first penetrative sexual experience. Sexual initiation before 16 years of age was defined as “Early SI” and then dichotomized (yes/no), which was similar to previous studies [24,46]. The following categories were used to assess their use of contraceptives during regular sexual contact: never, rarely, sometimes, often, and always. This variable was also subsequently recoded to give a binary variable “never or rarely use contraception,” with “yes” for the first two categories, and “no” for the last three categories. The question to assess reproductive health symptom(s) (“Did you have any of the following symptoms during the past 12 months?”) included the following items: (1) urethral or vaginal discharge, (2) painful urination, (3) genital inflammation, (4) genital ulcers, (5) genital itching, (6) genital herpes, and (7) hematuria or vaginal bleeding. A new binary variable for “reproductive health symptom(s)” was classified into the category “Yes” when at least one of seven items had a “yes” response; otherwise, it was “No.”

#### 2.2.3. Covariates

Covariates in this study included age (in years, continuous variable), sex (male/female), type of higher education (college/university), year of study (freshman/sophomore/junior/senior and above), area of residence (urban/rural), and geographic distribution of home (eastern/central/western). Area of residence was defined as whether the usual living district of participants before entering higher education was in a rural or urban area. Due to inter-regional inequality (e.g., regional disparities in economic development) and the relevant classification criteria of the National Bureau of Statistics of China [47], geographic distribution of home was divided into three categories: eastern, central and western.

### 2.3. Statistical Analysis

All statistical analyses were performed using Stata 17.0 (Stata Corp, College Station, TX, USA). Firstly, we applied Pearson’s *χ*^2^ tests to compare the distribution of sexual initiation, contraception, unintended health outcome, and basic characteristics among different numbers of siblings, which were categorized into four groups: none (only child), one sibling, two siblings, and three or more siblings. Secondly, logistic regression was used to identify whether number of siblings, only child, or birth order was associated with adolescent sexual and reproductive health. For adjustment, the socioeconomic factors of study participants (i.e., age, sex, geographic distribution of home, higher education type, and year of study) were included in all regressions. Odds ratios (ORs) with 95% confidence intervals (CIs) were reported, and the statistical significance was set at *p* < 0.05 with a two-tailed test.

## 3. Results

### 3.1. Characteristics of Study Participants

A total of 11,044 undergraduate students are included in this study, and Table 1 shows the SRH and basic characteristics of study participants by the number of siblings. Overall, the numbers of undergraduate students with no siblings, one sibling, two siblings and three or more siblings were 4803 (43.5%), 2902 (26.3%), 1393 (12.6%), and 1946 (17.6%), respectively.

The results show that 6.3% of only children had sexual initiation after drinking alcohol, significantly lower than that of students with siblings (*p* < 0.001). The prevalence of never or rarely using contraception increased for the only child group (9.4%), one-sibling group (12.6%), two-sibling group (12.9%), and three-or-more-sibling group (15.8%). Similarly, students with more siblings were more likely to report unprotected first intercourse, and the percent of students with no sibling, one sibling, two siblings, and three or more siblings was 13.2%, 16.3%, 17.9%, and 20.1%, respectively. In addition, 3.4% of students with no siblings ever had an unintended pregnancy, lower than 4.7% of students with one sibling, 5.0% of students with two siblings, and 6.1% of students with three or more siblings. Students with one sibling (31.5%), two siblings (34.8%), and three or more siblings (31.3%) reported higher rates of reproductive health symptom(s) than those with only one sibling (30.2%). Diagnosed STI(s) prevalence was similarly increased with the number of siblings (only child: 2.2%, one sibling: 2.3%, two siblings: 2.9%, three or more siblings: 3.1%).

Among the only child group, the one-sibling group, the two-sibling group, and the three-or-more-sibling group, the average age (mean ± SD) was 20.7 ± 0.43, 20.5 ± 0.71, 20.6 ± 1.48, and 20.5 ± 1.05, respectively. The proportion of males was lower than females across the four groups (*p* = 0.004), while the proportion of living in urban areas was highest in the one-child category (72.0%), second highest in the two-sibling category (49.7%), third highest in the one-sibling category (46.7%), and lowest in the three-or-more-sibling category (37.4%). The probability of only children in university was significantly higher than those of students with siblings (*p* < 0.001). Finally, 51.6% of only child homes were located in the economically developed eastern region, but the percentage of those with three or more siblings was only 40.5%.

### 3.2. Association of Sibship Size, Only Child, Birth Order, and SRH

The logistic estimates of associations between sibship size and SRH are shown in Table 2. Undergraduate students with more siblings were more likely to have sexual initiation after drinking alcohol (OR 1.055, 95% CI 1.007–1.105) or after using pornography (OR 1.070, 95% CI 1.000–1.146). As the number of siblings increased, the prevalence of never or rarely using contraception (OR 1.097, 95% CI 1.057–1.138) and unprotected first intercourse (OR 1.082, 95% CI 1.046–1.119) also increased. Moreover, more siblings were positively associated with unintended health outcomes, including unintended pregnancy (OR 1.087, 95% CI 1.027–1.152) and STI(s) diagnosis (OR 1.085, 95% CI 1.006–1.171).

Table 3 further reports whether being an only child or birth order is associated with SRH among Chinese young people. Being an only child was negatively related to sexual initiation after drinking alcohol (OR 0.818, 95% CI 0.698–0.957), never or rarely using contraception (OR 0.775, 95% CI 0.681–0.882), unprotected first intercourse (OR 0.797, 95% CI 0.711–0.892), and unintended pregnancy (OR 0.745, 95% CI 0.606–0.916). Among 6187 students with siblings, there was no significant difference between the youngest and the oldest children regarding sexual initiation, contraception, and unintended health outcomes. However, middle children were more likely to have sexual initiation after using pornography (OR 1.793, 95% CI 1.279–2.515) and to never or rarely use contraception (OR 1.407, 95% CI 1.169–1.693).

### 3.3. Stratified Analysis of Sibship Size for SRH

Table 4 displays the association of sibship size and SRH stratified by sex and area of residence. In terms of sex heterogeneity, both male and female students with more siblings were more likely to use contraception never or rarely (male: OR 1.090, 95% CI 1.033–1.151; female: OR 1.108, 95% CI 1.053–1.166) and have unprotected first intercourse (male: OR 1.105, 95% CI 1.053–1.159; female: OR 1.060, 95% CI 1.010–1.112). Furthermore, for male students, the prevalence of sexual initiation after drinking alcohol (OR 1.081, 95% CI 1.014–1.152) and unintended pregnancy (OR 1.104, 95% CI 1.016–1.200) increased as the number of siblings increased.

For students living in urban areas before university, there were more positive relationships between siblings with sexual initiation after drinking alcohol (OR 1.120, 95% CI 1.050–1.195), never or rarely using contraception (OR 1.111, 95% CI 1.050–1.176), unprotected first intercourse (OR 1.127, 95% CI 1.071–1.186), and reproductive health symptom(s) (OR 1.059, 95% CI 1.014–1.107). For students living in rural areas before university, more siblings were positively associated with sexual initiation after using pornography (OR 1.121, 95% CI 1.026–1.225), never or rarely using contraception (OR 1.084, 95% CI 1.031–1.138), unintended pregnancy (OR 1.083, 95% CI 1.003–1.168), and diagnosis of STI(s) (OR 1.119, 95% CI 1.014–1.235).

## 4. Discussion

Based on a large-scale national survey, this paper examined the association between the number of siblings, being an only child, birth order and SRH among young people in China, with indicators of SRH including risky sexual initiation, contraceptive use, and unintended health outcomes.

Generally, young people in China with more siblings were found to be related to more risky sexual initiation, less contraceptive use, and worse unintended health outcomes. For risky sexual initiation, our results indicate that youth with larger sibship size were more likely to have sexual initiation after drinking alcohol or using pornography. However, there was no significant association between more siblings and early sexual initiation (sexual debut before 16 years of age). In terms of less contraceptive use, having more siblings was related to higher odds of never or rarely using contraception and unprotected first intercourse. As a result of risky sexual initiation and less contraception, unintended pregnancy and being diagnosed with STI(s) were more common for students with more siblings, indicating poorer SRH. According to previous research, the SRH of young people may be negatively or positively affected by siblings. Competition for parental attention and resources results in negative effects [29,30], while instructions and communications regarding sexuality between siblings are often helpful in preventing risky sexual behaviors in young people [24,31,32]. Overall, our findings support that sibling rivalry was more prevalent among Chinese young people, and having more siblings was associated with risky sexual behaviors and negative SRH.

Notably, there were significant differences in the relationship between the number of siblings and SRH across sex groups and areas of residence before university. For males with more siblings, they were likely to have first sexual intercourse after drinking alcohol, never or rarely use contraception, and accidentally get their sexual partners pregnant. For females with more siblings, sibship size was mainly related to contraceptive use, including never or rarely using contraception, and unprotected sexual initiation, with no significant associations with risky sexual initiation or unintended health outcomes. Regardless of whether they lived in urban or rural areas, Chinese young people with more siblings had poorer SRH, but specific indicators differed. In urban youth with more siblings, sexual initiation after drinking alcohol, never or rarely using contraception, unprotected sexual debut, and reproductive health symptom(s) were more likely to occur. For students living in rural areas before university, larger sibship size meant higher likelihoods of sexual initiation after using pornography, never or rarely using contraception, and even severe health consequences, such as unintended pregnancy and diagnosis with STI(s). Our analysis supplemented the sibling effect in China, while the previous literature concentrated on physical health status [48,49] and academic achievements [18,50].

Young people in our sample were mainly born between 1996 and 2001, a period during which China implemented the one-child policy, and this gives us a precious opportunity to examine sibling effect on SRH, whether based on being the only children or birth order. The majority of the association between sibship size and SRH could be attributed to being an only child, which represents 43.5% of the total population. Compared to those of students with siblings, there were significant reductions in the odds of sexual initiation after drinking alcohol, sexual initiation after using pornography, never or rarely using contraception, unprotected first intercourse, and unintended pregnancy. One possible explanation is that only children have closer relationship with peers and parents than children with siblings. On the one hand, as a result of increased interactions with classmates and cousins, being an only child did not adversely affect SRH despite the absence of siblings [42]. On the other hand, in a family of three, the only child often has closer parent–child relationship, which also promotes the SRH of young people [21,22,23]. 

Studies conducted in the UK found that only child females were more likely to have first intercourse at later ages [25], and middle-child males were likely to have sexual initiation earlier compared to oldest children [25] or have first sexual intercourse under 16 [24]. This study found no significant correlation between early sexual initiation and being an only child, and last-borns were similar to first-borns in terms of sexual initiation, contraception, and unintended health outcomes. However, middle children had much higher odds of sexual initiation after using pornography and never or rarely using contraception than first-borns. In summary, Chinese only children had less risky sexual behaviors and better SRH than young people with siblings, while middle children suffered from less contraceptive use and risky sexual debuts. These findings offer contributions to research on family structure and SRH [21,22,23,24,25,26], demonstrating the importance of being only children and middle-borns in SRH.

To our knowledge, this study provides the first comprehensive evidence on the relationships between sibship size, being an only child, birth order, and the SRH of young people in China, but it suffers from several limitations as outlined below. Firstly, due to the cross-sectional study design, even with a large geographically dispersed sample and appropriate adjustment for socioeconomic factors, we were not able to establish the causal effect of siblings on SRH. Therefore, the causal effect of siblings on sexual reproduction and health requires further research. Secondly, self-reported data may introduce measurement errors due to recall bias and socially desirable manners, especially for SRH-related questions. In contrast, university students are more open-minded about sex. We have made great efforts to improve the confidentiality and anonymity of the survey and have implemented a social media survey method that allows respondents to respond in private which is also more suitable for sensitive matters. Finally, further studies are needed to investigate the potential mechanisms of variations in youth with different sibship size. Different sex composition of siblings is correlated with different sibling effects [51], for example, older sisters with greater levels of intimacy are very important for socialization [31], thus improving the SRH of only children. Age gaps between sibling may be another determinant, as the role of father monitoring could be strengthened when there is a large age gap between sisters [52].

Our findings have several implications for promoting SRH among Chinese young people. A long-standing social conservative attitude towards sex and a lack of formal sexuality education pose serious challenges to SRH, including false and violent sexual knowledge received from the Internet, high prevalence of risky sexual behaviors, and high rates of unintended pregnancy and abortion [17]. Based on the relationship between sibship size birth order and SRH in this study, sex education can be more efficient if targeted at different subgroups of Chinese young people. Specifically, effective prevention education, such as the promotion of condom use, is needed. In particular, risky sexual debuts and low frequency of contraception is prevalent among middle-borns. Since they often receive less attention and support from their parents, providing social support and improving parenting styles are as equally important. Furthermore, young people growing up in rural areas with more siblings are associated with severe SRH problems (e.g., unintended pregnancy and STI(s) diagnosis). Thus, sexual knowledge and awareness training are of greater importance for them, which contributes to the reduction in disease burden and promotes SRH throughout their lifetime.

## Figures and Tables

**Table 1:** Characteristics of study participants by the number of siblings.

	None (Only Child)		One		Two		Three or More		*χ* ^2^	*p*-Value
	*N*	%	*N*	%	*N*	%	*N*	%		
**Sexual initiation (SI)**										
SI after drinking alcohol	301	6.3	228	7.9	110	7.9	178	9.1	19.06	<0.001
SI after using pornography	160	3.3	67	2.3	43	3.1	87	4.5	17.70	0.001
Early SI	678	14.1	430	14.8	188	13.5	282	14.5	1.56	0.669
**Contraception**										
Never or rarely use contraception	453	9.4	367	12.6	180	12.9	307	15.8	58.96	<0.001
Unprotected first intercourse	635	13.2	474	16.3	250	17.9	392	20.1	56.88	<0.001
**Unintended health outcome**										
Unintended pregnancy	165	3.4	136	4.7	69	5.0	119	6.1	25.64	<0.001
Reproductive health symptom(s)	1450	30.2	913	31.5	485	34.8	609	31.3	10.80	0.013
Diagnosed STI(s)	108	2.2	68	2.3	40	2.9	60	3.1	5.04	0.169
**Covariates**										
Age (Mean SD)	20.7	0.43	20.5	0.71	20.6	1.48	20.5	1.05	18.05 ^1^	<0.001
Sex:									13.10	0.004
Male	2173	45.2	1227	42.3	573	41.1	813	41.8		
Female	2630	54.8	1675	57.7	820	58.9	1133	58.2		
Area of residence:									895.90	<0.001
Urban	3456	72.0	1355	46.7	693	49.7	727	37.4		
Rural	1347	28.0	1547	53.3	700	50.3	1219	62.6		
Type of higher education:									310.96	<0.001
College	960	20.0	929	32.0	448	32.2	765	39.3		
University	3843	80.0	1973	68.0	945	67.8	1181	60.7		
Year of study:									345.88	<0.001
Freshman	1141	23.7	972	33.5	461	33.1	793	40.7		
Sophomore	1051	21.9	668	23.0	345	24.8	514	26.4		
Junior	1007	21.0	534	18.4	236	16.9	326	16.8		
Senior and above	1604	33.4	728	25.1	351	25.2	313	16.1		
Geographic distribution of home:									134.01	<0.001
Eastern	2477	51.6	1312	45.2	658	47.3	788	40.5		
Central	1025	21.3	809	27.9	325	23.3	428	22.0		
Western	1301	27.1	781	26.9	410	29.4	730	37.5		

^1^ *F* statistics and *p*-value for ANOVA. STI(s), sexually transmitted infection(s).

**Table 2:** Logistic regression of sibship size for sexual and reproductive health among Chinese young people (*N* = 11,044).

	OR	S.E.	95% CI	Pseudo *R*^2^
**Sexual initiation (SI)**				
SI after drinking alcohol	1.055 *	0.025	(1.007,1.105)	0.026
SI after using pornography	1.070 *	0.037	(1.000,1.146)	0.022
Early SI	0.991	0.019	(0.953,1.029)	0.061
**Contraception**				
Never or rarely use contraception	1.097 ***	0.021	(1.057,1.138)	0.031
Unprotected first intercourse	1.082 ***	0.019	(1.046,1.119)	0.022
**Unintended health outcome**				
Unintended pregnancy	1.087 **	0.032	(1.027,1.152)	0.033
Reproductive health symptom(s)	1.026	0.016	(0.996,1.057)	0.080
Diagnosed STI(s)	1.085 *	0.042	(1.006,1.171)	0.015

STI(s), sexually transmitted infection(s). Adjusted for age, sex, geographic distribution of home, higher education type, and year of study. * *p* < 0.05, ** *p* < 0.01, *** *p* < 0.001.

**Table 3:** Logistic regression of one child or birth order for sexual and reproductive health among Chinese young people.

	Only Child (*N* = 11,044)		Birth Order (*N* = 6187)			
			Middle Child		Youngest Child	
	OR (S.E.)	95% CI	OR (S.E.)	95% CI	OR (S.E.)	95% CI
**Sexual initiation (SI)**						
SI after drinking alcohol	0.818 * (0.066)	(0.698,0.957)	0.890 (0.113)	(0.694,1.141)	1.001 (0.114)	(0.800,1.252)
SI after using pornography	1.154 (0.134)	(0.919,1.448)	1.793 *** (0.310)	(1.279,2.515)	0.793 (0.157)	(0.538,1.170)
Early SI	1.008 (0.060)	(0.896,1.133)	0.909 (0.093)	(0.744,1.110)	0.901 (0.084)	(0.750,1.082)
**Contraception**						
Never or rarely use contraception	0.775 *** (0.051)	(0.681,0.882)	1.407 *** (0.133)	(1.169,1.693)	1.071 (0.102)	(0.889,1.290)
Unprotected first intercourse	0.797 *** (0.046)	(0.711,0.892)	1.175 (0.102)	(0.991,1.393)	0.898 (0.077)	(0.760,1.062)
**Unintended health outcome**						
Unintended pregnancy	0.745 ** (0.078)	(0.606,0.916)	1.261 (0.190)	(0.939,1.694)	1.197 (0.172)	(0.903,1.587)
Reproductive health symptom(s)	0.919 (0.043)	(0.838,1.007)	1.083 (0.083)	(0.932,1.258)	0.996 (0.074)	(0.861,1.152)
Diagnosed STI(s)	0.873 (0.117)	(0.671,1.137)	1.344 (0.264)	(0.914,1.975)	1.014 (0.210)	(0.676,1.521)

STI(s), sexually transmitted infection(s). Reference group of birth order is oldest child. Adjusted for age, sex, geographic distribution of home, higher education type, and year of study. * *p* < 0.05, ** *p* < 0.01, *** *p* < 0.001.

**Table 4:** Logistic regression of sibship size for sexual and reproductive health among Chinese young people, stratified by sex or area of residence.

	Sex		Area of Residence	
	Male (*N* = 4786)	Female (*N* = 6258)	Urban (*N* = 4813)	Rural (*N* = 6231)
	OR (95% CI)	OR (95% CI)	OR (95% CI)	OR (95% CI)
**Sexual initiation (SI)**				
SI after drinking alcohol	1.081 * (1.014,1.152)	1.026 (0.958,1.099)	1.120 *** (1.050,1.195)	0.985 (0.920,1.055)
SI after using pornography	1.078 (0.988,1.176)	1.068 (0.956,1.194)	1.023 (0.919,1.139)	1.121 * (1.026,1.225)
Early SI	0.996 (0.944,1.051)	0.985 (0.932,1.042)	1.013 (0.961,1.068)	0.967 (0.914,1.023)
**Contraception**				
Never or rarely use contraception	1.090 ** (1.033,1.151)	1.108 *** (1.053,1.166)	1.111 *** (1.050,1.176)	1.084 ** (1.031,1.138)
Unprotected first intercourse	1.105 *** (1.053,1.159)	1.060 * (1.010,1.112)	1.127 *** (1.071,1.186)	1.044 (0.998,1.091)
**Unintended health outcome**				
Unintended pregnancy	1.104 * (1.016,1.200)	1.074 (0.991,1.163)	1.082 (0.989,1.183)	1.083 * (1.003,1.168)
Reproductive health symptom(s)	1.044 (0.989,1.101)	1.019 (0.984,1.056)	1.059 * (1.014,1.107)	0.997 (0.958,1.039)
Diagnosed STI(s)	1.119 (0.985,1.272)	1.065 (0.967,1.174)	1.003 (0.883,1.141)	1.119 * (1.014,1.235)

STI(s), sexually transmitted infection(s). Adjusted for age, sex, geographic distribution of home, higher education type, and year of study. * *p* < 0.05, ** *p* < 0.01, *** *p* < 0.001.

## Data Availability

The data are not publicly available due to conditions on participant consent and other ethical restrictions, as the data contain information that could identify and therefore compromise the privacy of research participants.
